# Are the tumor microenvironment characteristics of pretreatment biopsy specimens of colorectal cancer really effectively predict the efficacy of neoadjuvant therapy: A retrospective multicenter study

**DOI:** 10.1097/MD.0000000000039429

**Published:** 2024-08-30

**Authors:** Bingbing Li, Longjiao Chen, Yichun Huang, Meng Wu, Weilan Fang, Xin Zou, Yihong Zheng, Qiuxiang Xiao

**Affiliations:** a Department of Pathology, Ganzhou Hospital of Guangdong Provincial People’s Hospital, Ganzhou Municipal Hospital, Ganzhou, China; b Department of Pathology, Ganzhou People’s Hospital, Ganzhou, China; c Department of Pathology, The First Affiliated Hospital of Gannan Medical University, Ganzhou, China; d Department of Graduate School, China Medical University, Shenyang, China.

**Keywords:** colorectal cancer, neoadjuvant therapy, prognostic prediction, tumor microenvironment characteristics

## Abstract

More and more studies had pointed out that the tumor microenvironment characteristics based on colorectal cancer (CRC) pretreatment biopsy specimens could effectively predict the efficacy of neoadjuvant therapy, but under hematoxylin and eosin (HE) staining, whether the tumor microenvironment characteristics observed by pathologists could predict the efficacy of neoadjuvant therapy remains to be discussed. We collected 106 CRC patients who received neoadjuvant treatment and surgical resection from 3 hospitals. The number of mitosis, inflammation degree, desmoplastic reaction (DR), necrosis, tumor-stroma ratio (TSR) and tumor budding (TB) of CRC pretreatment biopsy specimens were observed under HE staining, and the degree of tumor pathological remission of CRC surgical specimens after neoadjuvant treatment was evaluated. According to the tumor regression grade (TRG), patients were divided into good-responders (TRG 0–1) and non-responders (TRG 2–3). All data were analyzed with SPSS software (version 23.0) to evaluate the correlation between the number of mitosis, inflammation degree, DR, necrosis, TSR and TB in pretreatment biopsy samples and the treatment effect. In univariate analysis, mitosis (*P* = .442), inflammation degree (*P* = .951), DR (*P* = .186), necrosis (*P* = .306), TSR (*P* = .672), and TB (*P* = .327) were not associated with the response to neoadjuvant therapy. However, we found that for colon cancer, rectal cancer was more likely to benefit from neoadjuvant therapy (*P* = .024). In addition, we further analyzed the impact of mitosis, inflammation degree, DR, necrosis, TSR and TB on neoadjuvant therapy in rectal cancer, and found that there was no predictive effect. By analyzing the characteristics of tumor microenvironment of CRC pretreatment biopsy specimens under HE staining, such as mitosis, inflammation degree, DR, necrosis, TSR and TB, it was impossible to effectively predict the efficacy of neoadjuvant therapy for CRC.

## 1. Introduction

Colorectal cancer (CRC) is the most common digestive system tumor worldwide, and currently surgical resection and adjuvant treatment are the main treatment methods.^[1]^ Preoperative neoadjuvant radiotherapy and chemotherapy are recommended for locally advanced rectal cancer patients. In addition, neoadjuvant chemotherapy has been introduced to treat unresectable CRC with oligometastasis.^[2]^ Compared to only undergoing surgical resection, neoadjuvant radiotherapy and chemotherapy also improve the overall survival and local recurrence free survival of some locally advanced CRC patients.^[3]^ There is still some heterogeneity in this type of patient, and the prognosis of each patient is different. Only 10% to 30% of patients with neoadjuvant radiotherapy and chemotherapy can achieve complete pathological remission.^[4]^ Therefore, in order to obtain better clinical results of neoadjuvant therapy, reduce the relative toxicity and side effects of neoadjuvant therapy, it is crucial to accurately screen patients who benefit or accurately evaluate the efficacy of neoadjuvant therapy before treatment.

At present, more and more studies show that the tumor microenvironment plays an important role in the tumor development, metastasis and treatment. For example, tumor-infiltration immune cells, cancer-related fibroblasts and tumor budding (TB) in the tumor microenvironment are prognostic indicators of various solid cancers,^[5–8]^ such as tumor-stroma ratio (TSR), desmoplastic reaction (DR), peritumoral inflammation, TB and other characteristics. Without other additional examinations, they can be observed and graded or scored under Haematoxylin and eosin (HE) staining. Although some studies have shown that TSR, tumor-infiltration immune cells, and TB in pretreatment biopsy samples can effectively predict the efficacy of neoadjuvant therapy,^[2,9,10,11]^ in addition, DR, tumor necrosis, and tumor cell mitosis have also been proven to be effective in predicting the prognosis of CRC .^[12–15]^ there are also some opposite research results,^[2,16]^ which may be that some methods have not been standardized. Therefore, it is very important to accurately evaluate the characteristics of tumor microenvironment in pretreatment biopsy samples and screen out effective prognostic indicators of neoadjuvant therapy. In this study, we observed TSR, DR, tumo-infiltration immune cells, tumor necrosis, tumor cell mitosis and TB in pretreatment biopsy samples under HE staining and graded or classified them, to evaluate whether the above tumor microenvironment characteristics can effectively predict the efficacy of neoadjuvant treatment, to verify the reliability of some conclusions of the current studies. In addition, this study is also a small-scale multicenter study, which provides a basis for screening the characteristics of tumor microenvironment predicted by neoadjuvant therapy.

## 2. Materials and methods

### 2.1. Patient information

We collected the clinical information and HE staining sections of 106 patients with CRC who received neoadjuvant therapy (neoadjuvant chemoradiotherapy or neoadjuvant chemotherapy) and underwent surgical resection in Ganzhou Municipal Hospital, The First Affiliated Hospital of Gannan Medical University and Ganzhou People’s Hospital in 2015 and 2021, including 17 patients in Ganzhou Municipal Hospital, 29 patients in The First Affiliated Hospital of Gannan Medical University, and 60 patients in Ganzhou People’s Hospital. The collected clinical pathological information mainly includes age, gender, histopathological type, tumor site, pretreatment clinical T, N, and M status, and clinical stage. All patients have received neoadjuvant chemoradiotherapy or neoadjuvant chemotherapy. The study was conducted in accordance with the Declaration of Helsinki, and approved by the Ethics Committee of Ganzhou Municipal Hospital (no. LW2024005H).

### 2.2. Analysis of histopathological characteristics

Pathologists were used to evaluate the tumor cell mitosis, inflammation degree, DR, tumor necrosis, TSR, and TB in pretreatment biopsy specimens under HE staining, and evaluate the neoadjuvant treatment response of tumor in surgical resection specimens. If there was disagreement between 2 pathologists during the evaluation process, a third pathologist were conduct a review to determine the final result.

#### 2.2.1. Number of tumor cell nuclear mitosis

**Figure 1.**
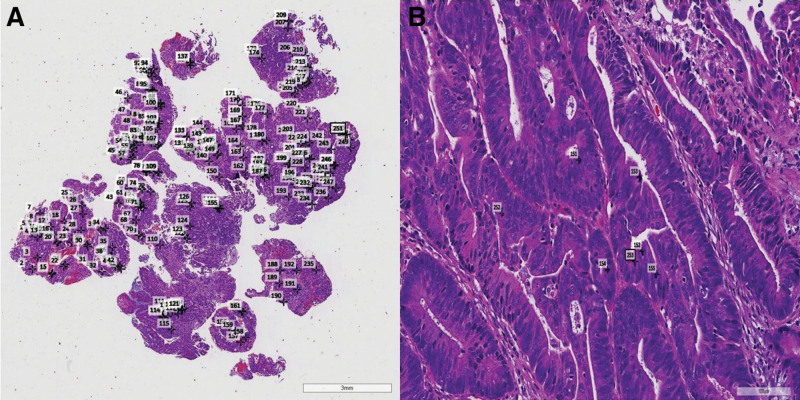
Representative image of tumor mitosis in pretreatment biopsy specimens of colorectal cancer. (A) Full field image of tumor mitosis labeled on WSI. (B) Tumor mitosis in hotspot areas, HE staining, 200 ×. WSI = whole slide image.

First, the pretreatment biopsy specimens of each patient were made into HE stained slides through neutral Formaldehyde fixation, paraffin embedding, sectioning, and HE staining processes, and then these slides were scanned through the full digital sectioning scanner, and then all tumor cell mitotic images were marked on the whole slide image (WSI) through imagescope software, Finally, selected 5 hotspots to count the total number of tumor cell nuclear mitotic images in that region (Fig. 1A and B).

#### 2.2.2. Inflammation degree

The inflammation degree mainly was designed to evaluate the invasive edge or infiltration front of the tumor in the surgical resection specimen and the degree of inflammatory cell response within tumor.^[17]^ This study mainly collected pretreatment biopsy specimens for neoadjuvant therapy. The biopsy specimens were often obtained through colonoscopy from the superficial tissue of the tumor, as this study was equivalent to evaluating the degree of inflammatory cell response within the tumor.

**Figure 2.**
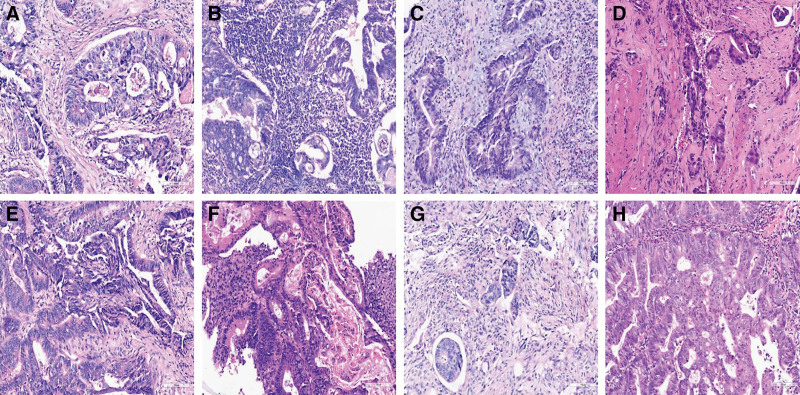
Representative images of tumor microenvironment in pretreatment biopsy specimens of colorectal cancer. (A) weak and (B) Strong grade inflammation degree. (C) immature DR and (D) mature Dr (E) no tumor necrosis and (F) tumor necrosis. (G) high and (H) low TSR. (A–H, HE staining, 200×). HE = Haematoxylin and eosin, TSR = tumor-stroma ratio.

We evaluated the degree of inflammatory response in pretreatment biopsy specimens according to the Klintrup–Mäkinen (KM) grading standard.^[17]^ The KM grade is semi-quantitatively scored using a four-grade scale. A score of 0 was given when there was no increase in the number of inflammatory cells; a score of 1 denoted a mild and patchy increase in inflammatory cells, but no destruction of invading cancer cell islets by the inflammatory cells; a score of 2 was given when inflammatory cells formed a band-like infiltrate, with some destruction of cancer-cell islets; and a score of 3 denoted a prominent inflammatory reaction, forming a cup-like zone, with frequent destruction of cancer-cell islets.^[17]^ Based on the KM grade method, pathologists observed all regions of WSI using imagescope software, then selected a hotspot, and finally evaluated the degree of inflammatory response within a 200-fold hotspot area. The inflammation degree was divided into two categories (Fig. 2A and B): weak (0 score and 1 score) and strong (2 score and 3 score).

#### 2.2.3. DR

DR was divided into 3 histological types: immature type, intermediate type, and mature type. The 3 types of DR was mainly distinguished according to the original mucoid matrix and Keloid-like collagen.^[18,19]^ Evaluated the composition of mucoid matrix and Keloid-like collagen at the front of tumor invasion under the microscope to determine the type of fibrogenic reaction. If the majority of them were mucoid matrix under the 400 field of vision or they were completely mucoid matrix and Keloid-like collagen coexisting under the 400 field of vision, they were immature DR type; If there was Keloid-like collagen in the fibrotic matrix without meeting the diagnostic criteria of immature DR type, it was intermediate DR type; If there was neither mucoid matrix nor Keloid-like collagen in the front of tumor invasion, it was mature DR type.

This study mainly focuses on pretreatment biopsy specimens. Considering the limited number of specimens, the DR types were mainly divided into 2 types (immature DR type and mature DR type). If there was a mucoid matrix in the tumor stroma in pretreatment biopsy tissue, it was considered an immature DR type; If there was no mucinous matrix in the tumor stroma in the pretreatment biopsy tissue, it was considered a mature DR type (Fig. 2C and D).

#### 2.2.4. Tumor necrosis

According to the previous research,^[12,15,20]^ tumor necrosis in HE stained sections was specified as an area with increased eosinophilia and nuclear shrinkage, fragmentation and disappearance, with shadows of tumor cells visible to variable extent. According to the above definition of tumor necrosis, pretreatment biopsy specimens were divided into 2 categories, namely with and without tumor necrosis (Fig. 2E and F).

#### 2.2.5. TSR

The definition of TSR was the percentage of stroma in tumor tissue,^[21–23]^ which could be directly measured under HE staining. As previously reported,^[24,25]^ slides were selected from the most invasive part of the tumor (i.e., The invasive front was chosen from the tissue block the pathologist selects as most invasive part and uses to determine the T-status). Used a 2.5× or 5× objective to select the area with the highest number of stroma and used a 10× objective to select the area with both tumor and stroma tissue, and then determine the final TSR score. Considering that the biopsy specimen have not the most invasive part (i.e. the slide used to determine the T state in routine pathology), the percentage of stroma in the tumor tissue was evaluated throughout the entire biopsy specimen. For further analysis, TSR was divided into 2 categories: low (≤50% stroma) and high (>50% stroma), as shown in Figure 2G and H.

#### 2.2.6. TB

**Figure 3.**
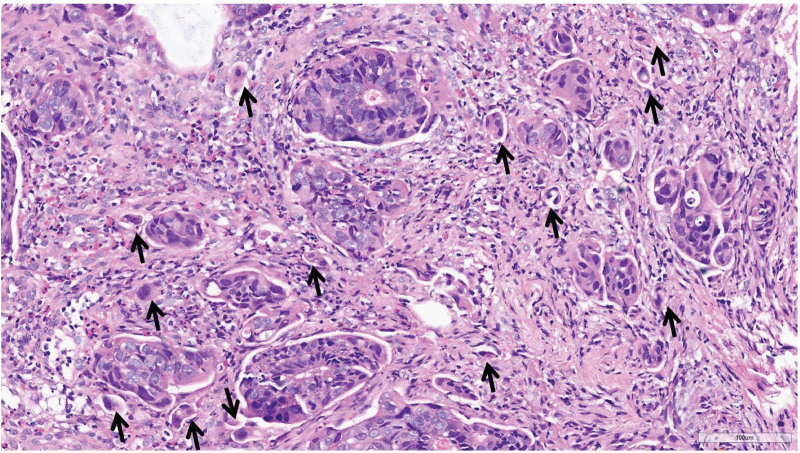
TB (arrow) in pretreatment biopsy specimens of colorectal cancer, HE staining, 200 ×. HE = Haematoxylin and eosin, TB = tumor budding.

TB^[26]^ was defined as single cells or clusters of up to 4 cells at the invasive margin of CRC, with an area of 0.785 mm^2^ or a field of 20× objective. The number of TB in the “hot spot area” was counted. If the number of TB was 0 to 4, it was considered a low-grade TB; The number of TB ranges from 5 to 9, indicated a moderate-grade TB; If the number of TB was 10 or more, it was considered a high-grade TB. Considering the limitations of biopsy specimens, this study adopts a 2 classification method for biopsy specimens, namely with or without tumor budding (Fig. 3).

#### 2.2.7. Evaluation of neoadjuvant therapy response

**Figure 4.**
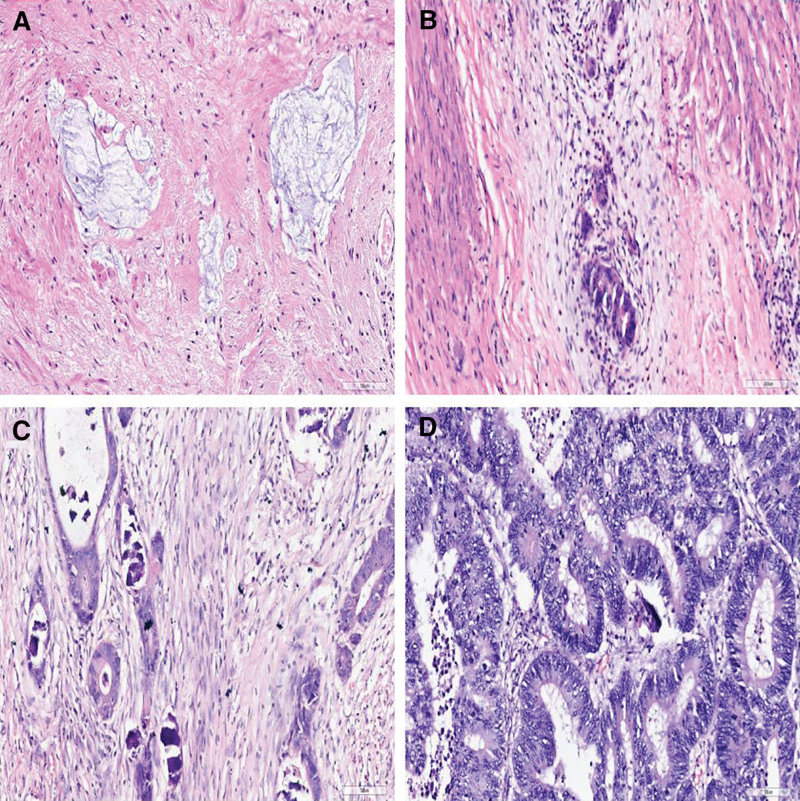
Representative images of TRG system. Responses of (A) TRG 0 and (B) TRG 1 categorized as good-responders. Responses of (C) TRG 2 and (D) TRG 3 were classified as non-responders. (A–D, HE staining, 200×). HE = Haematoxylin and eosin, TSR = tumor-stroma ratio.

The efficacy evaluation of neoadjuvant therapy response for surgical resection in this study, the main criteria used are the tumor regression grade (TRG) standards defined by the American Joint Commission on Cancer (AJCC, 8th edition). TRG 0 is defined as complete regression with no visible cancer cells and is called a pathological complete response. TRG 1 is characterized by single or small groups of tumor cells. TRG 2 is characterized by residual cancer outgrown by fibrosis. TRG 3 is defined by minimal or no tumor cells killed, Figure 4. According to TRG scores, neoadjuvant treatment reactions were divided into 2 categories, TRG 0 and TRG 1 were categorized as good-responders, whereas TRG 2 and TRG 3 were classified as non-responders.

### 2.3. Statistical analysis

The *t* tests were used to analyze the age, mitosis and other continuous factors, while the Pearson chi-squared test or Fisher exact test was used to test the classification characteristics. Logistic regression analysis was used to evaluate the independent significance of response predictors (defined as good-response or non-response): age, gender, clinical TNM stage, mitosis, DR, inflammation degree, tumor necrosis, TSR and TB.

All data analyses were conducted in SPSS statistical software (Windows version 23.0; SPSS Inc., Chicago). In all analyses, a two-tailed *P* value < .05 was regarded as statistically significant.

## 3. Results

### 3.1. Clinical and pathological characteristics of patients

In this study, a total of 106 CRC patients were recruited from 3 hospitals, with a median age of 58 years (standard deviation 10.16; range 25–85 years), of which 68 were male (64.15%) and 38 were female (36.85%); Among them, 71 cases were located in the rectum (66.98%), and 35 cases were located in the colon (33.02%); Among them, there were 100 cases of ordinary adenocarcinoma (94.34%) and 6 cases of mucinous adenocarcinoma (5.66%). The basic information and clinical pathological parameters of the patients were listed in Table 1. According to the TRG scores, 18 patients were classified as good-responders (16.98%), and 88 patients were classified as non-responders (83.02%); Previous studies had shown that only 10-30% of patients receiving neoadjuvant therapy benefit, while in this study, only 16.98% achieved good response to neoadjuvant therapy, consistent with relevant studies.

**Table 1:** Clinicopathological characteristics of patients.

	Total (n = 106)	Good-responders (n = 18)	Non-responders (n = 88)	*P*
Age (year, mean ± SD)	58.78 ± 10.16	60.78 ± 13.28	58.38 ± 9.44	.363
Gender				.768
Male	68 (64.2%)	11 (61.1%)	57 (64.8%)	
Female	38 (35.8%)	7 (38.9%)	31 (35.2%)	
Tumour location				**.007**
Colon	35 (33.0%)	1 (5.6%)	34 (38.6%)	
Rectum	71 (67.0%)	17 (94.4%)	54 (61.4%)	
Histopathological type				1.000
Adenocarcinoma	100 (94.3%)	17 (94.4%)	83 (94.3%)	
Mucinous adenocarcinoma	6 (5.7%)	1 (5.6%)	5 (5.7%)	
cT status				.667
cT2-3	54 (50.9%)	10 (55.6%)	44 (50.0%)	
cT4	52 (49.1%)	8 (44.4%)	44 (50.0%)	
cN status				.344
cN0	6 (33.3%)	40 (45.5%)	46 (43.4%)	
cN1-2	12 (66.7%)	48 (54.5%)	60 (56.6%)	
cM status				.170
M0	74 (69.8%)	15 (83.3%)	59 (67.0%)	
M1	32 (30.2%)	3 (16.7%)	29 (33.0%)	
cTNM status				**.053**
I+II	34 (32.1%)	5 (27.8%)	29 (33.0%)	
III	40 (37.7%)	11 (61.1%)	29 (33.0%)	
IV	32 (30.2%)	2 (11.1%)	30 (34.0%)	
Hospital				.694
Ganzhou Municipal Hospital	17 (16.0%)	4 (22.2%)	13 (14.8%)	
The First Affiliated Hospital of Gannan Medical university	29 (27.4%)	4 (22.2%)	25 (28.4%)	
Ganzhou People’s Hospital	60 (56.6%)	10 (55.6%)	50 (56.8%)	

Bold value indicates *P* < .10.

### 3.2. Tumor microenvironment characteristics of biopsy specimens

The pathologist evaluated the number of mitosis, inflammation degree, DR, tumor necrosis, TSR and TB on the WSI of biopsy specimens. The average number of mitosis in all biopsy specimens was 6.54/5HPF, with a median of 6/5HPF; The severity of weak and strong inflammation was 70 (66.04%) and 36 (33.96%), respectively; Among them, there were 33 cases of immature DR and 73 cases of mature DR; Among them, there were 60 cases with low TSR (≤50% stroma) and 46 cases with high TSR (>50% stroma); 70 cases had tumor necrosis, and 31 cases had TB. The specific results were shown in Table 2. The results showed that there was no significant difference in the above histological features between patients with good-response and non-response.

**Table 2:** Histomorphological characteristics of biopsy specimen.

	Total (n = 106)	Good-responders (n = 18)	Non-responders (n = 88)	*P*
Mitosis number (mean ± SD)	6.54 ± 5.66	6.78 ± 6.98	6.49 ± 5.40	.845
Inflammation degree				.951
Weak	70 (66.0%)	12 (66.7%)	58 (65.9%)	
Strong	36 (34.0%)	6 (33.3%)	30 (34.1%)	
Desmoplastic reaction				.181
Immature	33 (31.1%)	8 (44.4%)	25 (28.4%)	
Mature	73 (68.9%)	10 (55.6%)	63 (71.6%)	
Tumor necrosis				.303
Yes	70 (66.0%)	10 (55.6%)	60 (68.2%)	
No	36 (34.0%)	8 (44.4%)	28 (31.8%)	
Tumor-stroma ratio				.672
Low (≤50%)	60 (56.6%)	11 (61.1%)	49 (55.7%)	
High (>50%)	46 (43.4%)	7 (38.9%)	39 (44.3%)	
Tumor budding				.324
Yes	75 (70.8%)	11 (61.6%)	64 (72.7%)	
No	31 (29.2%)	7 (38.9%)	24 (27.3%)	

### 3.3. Predictors of neoadjuvant therapy response

Logistic regression analysis was used to evaluate the independent significance of clinical pathological factors and histological features of biopsy specimens as predictive indicators of complete remission (TRG 0–1) in TRG patients. Table 3 shown the 14 predictive factors analyzed (i.e., age, gender, tumor site, tumor type, cT, cN, cM, clinical stage, mitosis, inflammation degree, DR, TSR, tumor necrosis, and TB), but in univariate analysis, only 1 variable could predict the efficacy of neoadjuvant therapy. This study found that rectal cancer was more likely to benefit from neoadjuvant therapy (*P* = .024). Could the number of mitosis, inflammation degree, DR, TSR, tumor necrosis, and TB in pretreatment biopsy specimens predict the efficacy of neoadjuvant therapy for rectal cancer? For further analysis, all cases of rectal cancer were selected, total 71 cases. Using univariate logistic regression analysis, it was found that all the above characteristics could not predict the efficacy of neoadjuvant therapy for rectal cancer (*P* > .05), as shown in Table 4.

**Table 3:** Univariable analysis with the logistic regression in colorectal cancer patients.

	OR (95% CI)	*P*
Age		
<60	Reference	
≥60	0.637 (0.230–1.767)	.386
Gender		
Male	Reference	
Female	0.855 (0.301–2.427)	.768
Tumour location		
Colon	Reference	
Rectum	0.093 (0.012–0.734)	**.024**
Histopathological type		
Adenocarcinoma	Reference	
Mucinous adenocarcinoma	1.024 (0.112–9.331)	.983
cT status		
cT2-3	Reference	
cT4	1.250 (0.451–3.464)	.668
cN status		
cN0	Reference	
cN1-2	0.600 (0.207–1.742)	.348
cM status		
M0	Reference	
M1	2.458 (0.659–9.171)	.181
cTNM status		
I+II	Reference	
III	0.455 (0.140–1.473)	.189
IV	2.586 (0.464–14.406)	.278
Mitosis number		
≤6.5/5HPF	Reference	
>6.5/5HPF	1.520 (0.523–4.418)	.442
Inflammation degree		
Weak	Reference	
Strong	1.034 (0.353–3.030)	.951
Desmoplastic reaction		
Immature	Reference	
Mature	2.016 (0.713–5.696)	.186
Tumor necrosis		
Yes	Reference	
No	0.583 (0.208–1.638)	.306
Tumor-stroma ratio		
Low (≤50%)	Reference	
High (>50%)	1.251 (0.444–3.527)	.672
Tumor budding		
Yes	Reference	
No	1.697 (0.589–4.885)	.327

Bold value indicates *P* < .05.

**Table 4:** Univariable analysis with the logistic regression in rectal cancer patients.

	OR (95% CI)	*P*
Age		
<60	Reference	
≥60	0.650 (1.960–0.216)	.444
Gender		
Male	Reference	
Female	0.714 (2.188–0.233)	.556
Histopathological type		
Adenocarcinoma	Reference	
Mucinous adenocarcinoma	0.615 (7.239–0.052)	.699
cT status		
cT2–3	Reference	
cT4	1.406 (4.196–0.471)	.541
cN status		
cN0	Reference	
cN1–2	0.562 (1.818–0.174)	.336
cM status		
M0	Reference	
M1	2.885 (14.160–0.588)	.192
cTNM status		
I+II	Reference	
III	0.505 (1.735–0.147)	.278
IV	4.444 (42.175–0.468)	.194
Mitosis number		
≤6.5/5HPF	Reference	
>6.5/5HPF	1.467 (4.542–0.474)	.507
Inflammation degree		
Weak	Reference	
Strong	0.917 (2.879–0.292)	.882
Desmoplastic reaction		
Immature	Reference	
Mature	1.820 (5.661–0.585)	.301
Tumor necrosis		
Yes	Reference	
No	0.563 (1.703–0.186)	.309
Tumor–stroma ratio		
Low (≤50%)	Reference	
High (>50%)	1.467 (4.542–0.474)	.507
Tumor budding		
Yes	Reference	
No	2.208 (0.699–6.971)	.177

## 4. Discussion

This study found that in neoadjuvant therapy for CRC, rectal cancer was more likely to benefit compared to colon cancer. In addition, for CRC, the number of mitosis, inflammation degree, tumor necrosis, DR, TSR, and TB in pretreatment biopsy specimens could not effectively predict the efficacy of neoadjuvant therapy for CRC. As far as we known, this was the first small scale multicenter study to evaluate whether the characteristics of tumor microenvironment (i.e., mitosis, inflammation degree, tumor necrosis, DR, TSR, and TB) in pretreatment biopsy specimens could predict the efficacy of neoadjuvant therapy for CRC under HE staining. The previous studies mainly focused on rectal cancer. In addition, most studies needed to use additional detection methods,^[10,11,27,28]^ Such as immunohistochemistry technology, immunofluorescence technology, and artificial intelligence algorithms.

In recent years, most studies had shown that CRC patients who benefit from neoadjuvant therapy often have better prognosis. Therefore, how to accurately and effectively predict the efficacy of neoadjuvant therapy for CRC has always been a challenge. we investigated relevant studies, and found that most studies showed that the tumor microenvironment in pretreatment biopsy samples could predict the efficacy of neoadjuvant therapy for rectal cancer. For example, El et al^[11]^ calculated the immune score by evaluating the average density of CD3+ and CD8+ T cells in pretreatment biopsy samples of rectal cancer, and found that patients with high immune scores in pretreatment biopsy samples had better efficacy of neoadjuvant therapy; Liang et al^[3]^ classified the TSR in pretreatment biopsy specimens of rectal cancer into 3 or 2 categories based on artificial intelligence semi-automatic methods. When the stroma content in pretreatment biopsy specimens is high, patients receiving neoadjuvant treatment often have poor response (TRG 2–3); Wen et al^[9]^ found that rectal cancer patients with high-grade TB or immature DR type in pretreatment biopsy specimens showed poor response to neoadjuvant therapy. In addition, some scholars believed that some tumor microenvironment characteristics could not predict the efficacy of neoadjuvant therapy. For example, research by Santos et al^[16]^ showed that there was a lack of effective histological characteristics in pretreatment biopsy specimens to predict the efficacy of neoadjuvant therapy for rectal cancer, such as mitotic index, necrosis grade, desmoplastic infiltration and inflammatory reaction grade could not play a predictive role; Yim et al^[2]^ also proved that the characteristics of immature DR, TSR, and KM grade in pretreatment biopsy specimens could not predict the treatment response or prognosis of rectal cancer after neoadjuvant therapy. Based on the contradiction of the above views, we carried out this small-scale multi center study to explore whether the tumor microenvironment characteristics of CRC for biopsy specimens could effectively predict the efficacy of neoadjuvant therapy?

Unfortunately, we had not found very valuable tumor microenvironment characteristics in pretreatment biopsy specimens to predict the efficacy of neoadjuvant therapy. This study mainly used previous research methods or recognized methods to evaluate the characteristics of tumor microenvironment, as described in the introduction of methods. In addition, in order to reduce the wrong classification or score caused by subjective factors, observation fatigue or other reasons when pathologists observe these characteristics of tumor microenvironment, In this study, 2 pathologists evaluated the characteristics of tumor microenvironment in these pretreatment biopsy samples. If there were differences between them, senior pathologists were review them to determine the final results. Therefore, the results of this study were reliable. Although our findings were consistent with some research results, in order to better screen valuable and repeatable tumor microenvironment features, we speculated on the screening and evaluation methods of tumor microenvironment features based on our research results and previous research findings. For mitosis, inflammation degree, DR and TB, these characteristics were closely related to tumor evolution and invasion behavior, so it was necessary to evaluate them by observing the tumor invasion front.^[13,16,17,19,29]^ Pretreatment biopsy samples were often shallow, so it was impossible to obtain infiltration front components, so the predictive performance of mitosis, inflammation degree, DR and TB may be poor. For TSR, surgical resection specimens were evaluated based on the deepest infiltrated tissue, while for the entire tumor specimen, biopsy tissue also has a certain degree of randomness. Therefore, it was not possible to exclude the possibility of contingency in the predictive role of TSR (such as Hansen et al^[30]^ found that the higher the proportion of stroma in surgical resection specimens after neoadjuvant therapy, the worse the prognosis of colon cancer patients; at the same time, this study compared TSR in biopsy specimens and surgical resection specimens, with 52 patients with low stroma content in biopsy specimens and only 33 patients with surgical resection specimens.), But more research is needed to verify whether TSR really has a predictive effect on neoadjuvant therapy in biopsy specimens. The limitations of biopsy specimens may also be the reason why tumor necrosis is ineffective in predicting neoadjuvant therapy. Therefore, it may be accidental to predict the efficacy of neoadjuvant therapy for CRC only by evaluated the characteristics of tumor microenvironment in pretreatment biopsy specimens under HE staining, but this was only speculation, and further big data multi center research is needed to verify whether this view is correct.

This study also had certain limitations, First, although this study used multicenter data, the number of cases was too small to further validate the above viewpoint; Second, this study was based on the TRG classification to determine the role of tumor microenvironment characteristics in predicting the efficacy of neodjuvant treatment, rather than through OS or DFS, because we could ot obtain follow-up data of patients; Third, although the previous evaluation methods were used to evaluate the characteristics of tumor microenvironment, some of them still had minor changes, mainly because of the small sample. For example, we only divided it into the presence or absence of TB, and did not further divide TB into low, medium and high grades. In future research, with the accumulation of samples, we will further refine these tumor microenvironment to further verify these conclusions.

## 5. Conclusions

This study further explored and found that the characteristics of tumor microenvironment based on pretreatment biopsy specimens might not be able to effectively screen CRC patients who can benefit from neodjuvant treatment, but for colon cancer patients, rectal cancer patients were more likely to benefit from it.

## Author contributions

**Conceptualization:** Bingbing Li, Longjiao Chen, Yichun Huang, Meng Wu, Yihong Zheng, Qiuxiang Xiao.

**Data curation:** Bingbing Li, Longjiao Chen, Yichun Huang, Meng Wu, Weilan Fang, Xin Zou, Yihong Zheng, Qiuxiang Xiao.

**Formal analysis:** Bingbing Li, Longjiao Chen, Yichun Huang, Meng Wu, Yihong Zheng, Qiuxiang Xiao.

**Funding acquisition:** Bingbing Li, Yihong Zheng, Qiuxiang Xiao.

**Investigation:** Bingbing Li, Longjiao Chen, Yichun Huang, Meng Wu, Weilan Fang, Xin Zou, Yihong Zheng, Qiuxiang Xiao.

**Methodology:** Bingbing Li, Longjiao Chen, Yichun Huang, Meng Wu, Yihong Zheng, Qiuxiang Xiao.

**Project administration:** Bingbing Li, Qiuxiang Xiao.

**Resources:** Bingbing Li, Yihong Zheng, Qiuxiang Xiao.

**Supervision:** Bingbing Li, Qiuxiang Xiao.

**Validation:** Bingbing Li.

**Visualization:** Bingbing Li.

**Writing – original draft:** Bingbing Li, Longjiao Chen, Yichun Huang, Meng Wu, Weilan Fang, Xin Zou, Yihong Zheng, Qiuxiang Xiao.

**Writing – review & editing:** Bingbing Li, Qiuxiang Xiao.
